# Unlocking the potential of *Kluyveromyces marxianus* in the definition of aroma composition of cheeses

**DOI:** 10.3389/fmicb.2024.1464953

**Published:** 2024-09-18

**Authors:** Giorgia Perpetuini, Alessio Pio Rossetti, Arianna Rapagnetta, Rosanna Tofalo

**Affiliations:** Department of Bioscience and Technology for Food, Agriculture and Environment, University of Teramo, Teramo, Italy

**Keywords:** *Kluyveromyces marxianus*, aroma compounds, OAV, qPCR, cheese

## Abstract

**Introduction:**

The cheese microbiota is very complex and is made up of technologically-relevant, spoilage, opportunistic and pathogenic microorganisms. Among them lactic acid bacteria and yeasts are the main ones. One of the most interesting dairy yeasts is *Kluyveromyces marxianus* because of its technological properties including the ability to produce aroma compounds.

**Methods:**

This study investigated the contribution of *Kluyveromyces marxianus* to the gross composition and aroma profile of cow cheeses. Experimental cheeses were prepared by inoculating a co-culture of *K. marxianus* FM09 and a commercial strain of *Lacticaseibacillus casei* and compared with cheeses obtained with only *L. casei*. The gross composition was determined by a FoodScan™ 2 Dairy Analyser, and free amino acids were evaluated at 507 nm after reaction with Cd-ninhydrin. The volatile organic compounds were extracted by head-space solid phase micro-extraction and analyzed by gas chromatography–mass spectrometry coupled with odor activity values. qRT-PCR was applied to determine the expression of genes involved in esters synthesis and degradation.

**Results:**

The inoculation of *K. marxianus* induced an increase of pH and a reduction of protein content of cheeses, in agreement with the stronger proteolysis detected in these cheeses. *K. marxianus* influenced the content of aroma compounds both quantitatively and qualitatively. In particular, an increase of higher alcohols, esters and organic acids was observed. Moreover, 12 compounds were detected only in cheeses obtained with the co-culture. These differences were in agreement with the odor activity values (OAV). In fact, only 11 compounds showed OAV > 1 in cheeses obtained with the commercial strain, and 24 in those obtained with the co-culture. The qPCR analysis revealed an over expression of *ATF*1, *EAT*1, and *IAH*1 genes.

**Conclusion:**

*Kluyveromyces marxianus* could act as an important auxiliary starter for cheese production through the development and diversification of compounds related to flavor in short-aged cow cheeses.

## 1 Introduction

The global cheese production is consistently growing, with the global cheese market expected to reach $287.12 billion by 2032, increasing from $191.94 billion in 2024, at a compound annual growth rate (CAGR) of 4.61% during the forecast period. The dairy industry has to meet the growing consumer demand for dairy products with unique flavors. Cheese flavor is one of the most relevant attributes influencing consumers’ acceptance and preference (Arora et al., 1995). The taste and aroma of cheese are determined by a complex combination of volatile and non-volatile components. Each compound contributes to the overall flavor, but cannot fully represent it on its own (Padilla et al., 2014).

The characteristics of cheese are influenced by several factors, including the type of milk used, the diet of the animals, the ripening conditions, the length of ripening, and the microbiota (Mayo et al., 2021). Microorganisms play a crucial role in the fermentation process and in the biochemical reactions that take place during the manufacturing and ripening processes. The cheese microbiota consists of a diverse group of microorganisms, including prokaryotes, eukaryotes, and viruses. Among them, lactic acid bacteria (LAB) are the most abundant and play a significant role in the production and aging of cheese. However, yeasts represent an important component of the microbiota of several cheese varieties (Bintsis, 2021). The occurrence of yeasts in these products is related to their ability to develop at low temperatures, assimilate/ferment lactose, and assimilate organic acids, besides proteolytic and lipolytic activities and resistance against high salt concentrations (Bintsis, 2021; Padilla et al., 2014). Yeasts are involved in the increase in pH on the cheese surface, which is caused by the metabolization of lactate into CO_2_ and H_2_O; and they use amino acids as energy sources after lactate exhaustion, producing considerable amounts of ammonia (Padilla et al., 2014). Nevertheless, yeasts can also induce cheese deterioration through early gas production, creating numerous small holes. Yeasts may also cause pink discoloration, brown patches, and deacidification of the cheese surface when they catabolize amino acids to produce NH_3_ (Bintsis, 2021; Zheng et al., 2021).

One of the most interesting dairy yeasts is *Kluyveromyces marxianus*. This yeast is thermotolerant, since it has been shown to grow at temperatures as high as 42°C. However, it is also able to develop at refrigerated temperatures (Fonseca et al., 2008; Tofalo et al., 2014). *Kluyveromyces marxianus* strains affect the ripening of cheese by their proteolytic and lipolytic activities, as well as the formation of volatile aroma compounds (Binetti et al., 2013; Padilla et al., 2014). This yeast species metabolizes lactose as carbon source due to the expression of the *LAC*12 and *LAC*4 genes, which code for a lactose permease and a *β*-galactosidase, respectively (Varela et al., 2017). The strains metabolize the lactose to generate ethanol and carboxylic acids, which are then converted into esters (resulting in a fruity flavor) and acetaldehyde. *K. marxianus* has been linked to the development of acidic, cidery, alcoholic, fermented, and fruity flavors (Zheng et al., 2021). This species has also acquired the QPS (Qualified Presumption of Safety) and GRAS (Generally Recognized as Safe) status from the European Food Safety Authority (EFSA) (EFSA Panel on Additives and Products or Substances used in Animal Feed [FEEDAP], 2004; Wang et al., 2018; Perpetuini et al., 2020).

As stated above, yeasts have a beneficial effect on the development of cheese aroma, which has generated commercial interest in utilizing specific strains as starter cultures together with LAB (Fröhlich-Wyder et al., 2019). In this study the effect of *K. marxianus* on cow cheese aroma profile was investigated. *K. marxianus* FM09 was used to make experimental cheese, with cheese made using a LAB commercial starter being used as the control group. The gross composition and the volatile profile of the cheeses were determined. Moreover, the expression of genes involved in esters synthesis and degradation in *K. marxianus* was evaluated.

## 2 Materials and methods

### 2.1 Strains used in this study

A commercial LAB strain of *Lacticaseibacillus casei* (CC) and the *K. marxianus* FM09 strain were used. *K. marxianus* FM09 was isolated from fermented milk (Fasoli et al., 2016). FM09 and CC strains were routinely grown in YPD or MRS, respectively. Strains were stored at −80°C in YPD or MRS broth supplemented with glycerol (20% v/v final concentration). Both strains belong to the Culture Collection of the Microbial Biotechnology Laboratory (University of Teramo, Teramo, Italy).

### 2.2 Cheesemaking procedure

Cheeses were manufactured on a laboratory scale under aseptic conditions. The cow pasteurized milk (72°C for 15 s) was distributed in 5 L containers. Three cheesemaking trials were carried out in 3 different days using the milk collected that day. In each trial, two cheese batches were simultaneously produced using two different starter combinations: the first inoculating only the CC and the second with a co-culture of CC and *K. marxianus* FM09. Therefore, a total of 6 trials were obtained, 3 inoculated with CC and 3 with CC + FM09. To prepare the inoculum for cheesemaking, the strains were inoculated in pasteurized cow milk incubated at 30°C for 48 h and inoculated at a final 7 Log CFU/mL concentration. A commercial bovine rennet was added according to the manufacturer’s instructions. After 15 min at 37°C the curd was cut and the whey was eliminated. The containers were stored at 15°C for 30 days.

### 2.3 Gross composition of cheeses

The main chemical characteristics (moisture, fat, and protein) were determined by a FoodScan™ 2 Dairy Analyser (Foss, Padova, Italy). The pH was determined by the pHmeter (Mettler Toledo, Milan, Italy) and the Aw was determined by Water Lab (Steroglass, Perugia, Italy) in agreement with the manufacturer’s instructions. Free amino acids (FAAs, expressed as mg leucine/g) were evaluated at 507 nm after reaction with Cd-ninhydrin according to Folkertsma and Fox (1992). Three technical replicates were performed on each sample.

### 2.4 Microbiological analysis

For the microbiological analysis, serial dilutions in sodium citrate (2% w/v) were prepared starting from 10 g of cheese. In both trials yeasts and LAB were enumerated. Yeasts were enumerated on Yeast Peptone Dextrose Agar (YPD; 1% w/v yeast extract, 2% w/v peptone, 2% w/v glucose and 2%w/v agar; Oxoid, Milan, Italy) supplemented with chloramphenicol (150 mg/L) at 28°C for 2 days, while LAB on DeMan-Rogosa-Sharp Agar (MRS) (Oxoid), acidified to pH 5.4 with acetic acid, and supplemented with 100 ppm cycloheximide (Merk Life Science S.r.l., Milan, Italy). Plates were incubated at 30°C for 2 days in anaerobic conditions using the Gas-Pack anaerobic system (AnaeroGen; Oxoid). Cell counts were performed in triplicate on each sample.

### 2.5 Determination of aroma compounds

Solid-phase microextraction (SPME) was used to extract volatile organic compounds (VOCs) according to Li et al. (2022). The gas chromatography–mass spectrometry (GC–MS) analysis was carried out using a gas chromatograph (Clarus 580; Perkin Elmer, Waltham, MA, United States) coupled with a mass spectrometer (SQ8S; Perkin Elmer). The gas chromatograph was equipped with an Elite-5MS column (length × internal diameter: 30 × 0.25 mm; film thickness: 0.25 μm; Perkin Elmer). An 85 μm fiber coated with carboxen-polydimethylsiloxane (Sigma-Aldrich, Milan, Italy) was used. Aroma components were identified by comparing the retention times of pure reference standards tested under the same conditions. The following standards were selected according to Sandoval-Copado et al. (2018) and Padilla et al. (2014): diacetyl, 2,3-pentanedione, 2-butanone, 2-nonanone, 2-hexanone, acetic acid, lactic acid, butyric acid, isovaleric acid, valeric acid, acetoin, propionic acid, decanoic acid, hexanoic acid, furfural, acetaldehyde, decanal, butyraldehyde, *δ*-decalactone, *γ*-dodecalactone, δ-dodecalactone, 1-octanol, 2,3-butanediol, 2-phenylethanol, isoamyl alcohol, 1-hexanol, ethyl hexanoate, phenylethyl acetate, isoamyl acetate, ethyl octanoate, diethyl succinate, ethyl acetate, ethyl butanoate. The standard solutions were purchased from Merk Life Science S.r.l (Mila, Italy) and were of the highest purity available. 2-Methyl-hexanol was used as internal standard. A minimum similarity criterion of 85% was used to compare mass spectra with MS fragmentation patterns in the National Institute for Standards and Technology database (NIST version 2005) in order to identify the compounds provisionally. The volatile compounds were quantified using the calibration curve of standards. The analyses were performed in triplicate. The odor activity values (OAV) were used to describe the intensity of odor compounds in the sample. The OAV is the ratio of a single compound’s concentration to that compound’s odor threshold. When a compound has OAV *>* 1, it is considered to contribute to the overall aroma of the sample. The analyses were performed in triplicate.

### 2.6 qRT-PCR analysis

RNA was extracted using RNeasy PowerSoil Total RNA Kit (Qiagen, Mila, Italy) according to the manufacturer’s instructions in order to determine the expression of the following genes: *ATF*1, *EAT*1, and *IAH*1. RNA concentration was determined spectrophotometrically with a Nanodrop spectrophotometer ND-1000 and 1 μg total RNA was retrotranscribed into cDNA, using the iScript cDNA Synthesis Kit (Bio-Rad) according to the manufacturer’s instructions. qRT-PCR was performed according to Muñoz-Miranda et al. (2024). Briefly, the following primer pairs were used:

IAH1_F 5’ CAGCGGAAAGGGTTACGAAG 3′ and IAH1_R 5’ AGGGGTCAAGAGTAGAGCCA 3′, ATF1_F 5’ AAAATCCCAAG AAACGAACCCA 3′ and ATF1_R 5’ TACGGCATCTCTGTCCCCG 3′, EAT1_F 5’ GCCCTTGTGAAATCTTGCGTT 3′ and EAT1_R 5’ CCGAGGCTTTGTGTCTCCATAG 3′. The qRT-PCR mixture (25 μL) contained: 12.5 μL SYBR™ Green PCR Master Mix (Thermo Fisher Scientific, Milan, Italy), 1 μL of template (100 ng/μL), 0.25 μL of each primer (10 μM) and diethylpyrocarbonate-treated water (DEPC-water). The thermal cycling conditions were as follows: 95°C for 10 min, 40 cycles of 30 s at 95°C, and 60 s at 60°C. *ALG*9 (coding for alpha-1,2-mannosyltransferase, ALG9_F 5’ CCATCTCAGGATCCC TCTTC 3′, ALG9_R GCATTCCAGCGAATAGTTGA 3′) and *ACT*1 (coding for actin, ACT1_F 5’ GGCTGAACGTGGTTACTCCT 3′ and ACT1_R 5’ AGAAGCGGTTTGCATTTCTT 3′) were used as reference genes (Perpetuini et al., 2019). qRT-PCR was performed using an iCycler IQ realtime PCR Detection System (Bio-Rad, Milan, Italy). Melting temperature analysis of the PCR products was performed to determine the specificity of the PCR reactions. Calculation of relative transcript levels (RTLs) was carried out using the comparative *C*t method [2–ΔCt gene – ΔCt reference gene], as described by Livak and Schmittgen (2001). All analyses were performed in triplicate.

### 2.7 Statistical analysis

Prism 7.0 program (GraphPad Software Inc., La Jolla, CA, United States) was used to analyze data and prepare graph. Results were expressed as mean value ± standard deviation. t-test was used to determine significance (*p* < 0.05) concerning the gross composition and volatile compounds. In order to avoid increased chance of false positive for the comparison between CC and FM09 + CC, multiple comparison correction, accounting for the main volatile compounds, was performed through the False Discovery Rate (FDR) algorithm (Genovese et al., 2002).

## 3 Results

### 3.1 Gross composition and microbial count of cheeses

**Figure 1:**
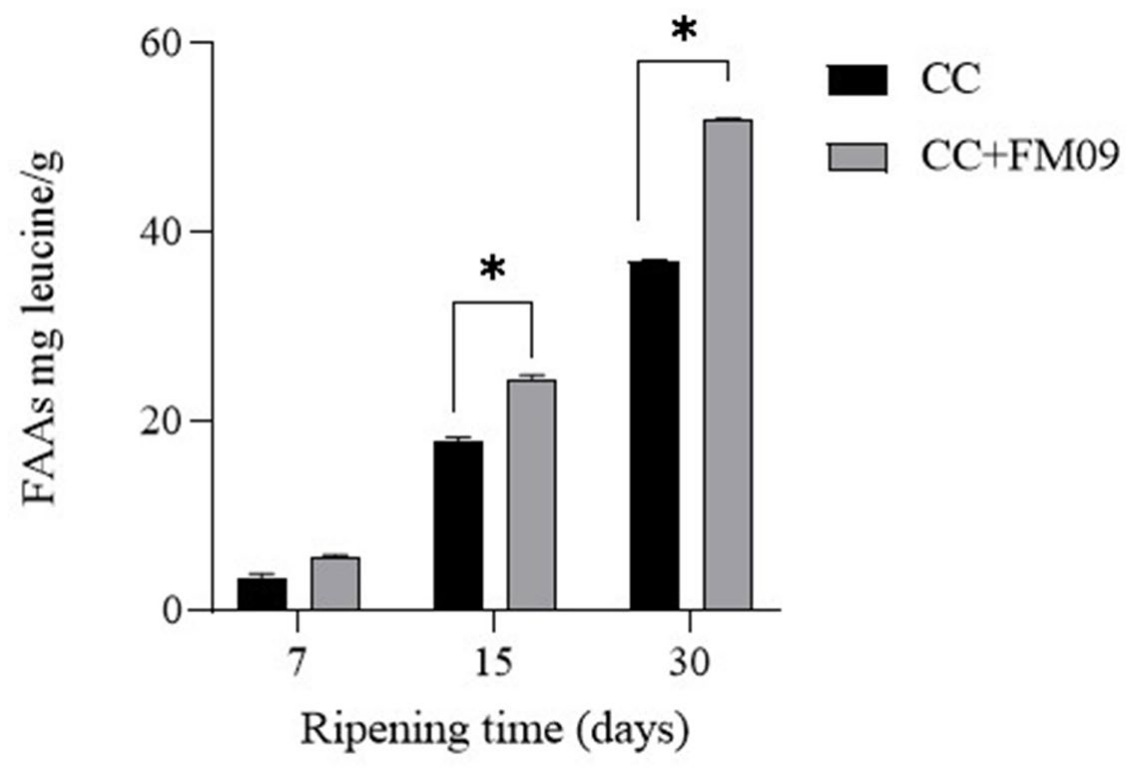
Evolution of free amino acids throughout ripening in cheeses produced with the commercial starter (CC) and CC + *K. marxianus* FM09. Data are means ± standard deviations of 3 independent experiments, each carried out in triplicate. * *p*<0.05.

The physico-chemical composition of cheeses is reported in Table 1. The main differences were detected for the pH and the protein content. In particular, *K. marxianus* FM09 induced an increase of pH, probably because of the lactic acid utilization by this yeast, and a reduction of protein content in agreement with the stronger proteolysis detected in these cheeses (Figure 1). Figure 1 showed the evolution of FAAs during the ripening period. The FAAs content increased significantly during ripening reaching a concentration of 36.98 mg leucine/g and 51.87 mg leucine/g in cheeses obtained with CC and CC + FM09, respectively.

**Table 1:** Physico-chemical characteristics of cheeses after 30 days of ripening.

Parameter	CC	CC + FM09
pH	5.35 ± 0.12^A^	5.81 ± 0.88^B^
Aw	0.96 ± 0.05^A^	0.96 ± 0.07^A^
Moisture	40.35 ± 2.42^A^	42.25 ± 1.99^A^
Fat (%)	25.15 ± 1.89^A^	26.61 ± 3.45^A^
Protein (%)	22.13 ± 3.15^A^	19.17 ± 0.89^B^

Different letters in the same line indicate significant differences (*p* < 0.05, FDR corrected). Data are means ± standard deviations of 3 independent experiments, each carried out in triplicate.

The addition of *K. marxianus* FM09 did not influence the other parameters which did not show significant differences.

**Figure 2:**
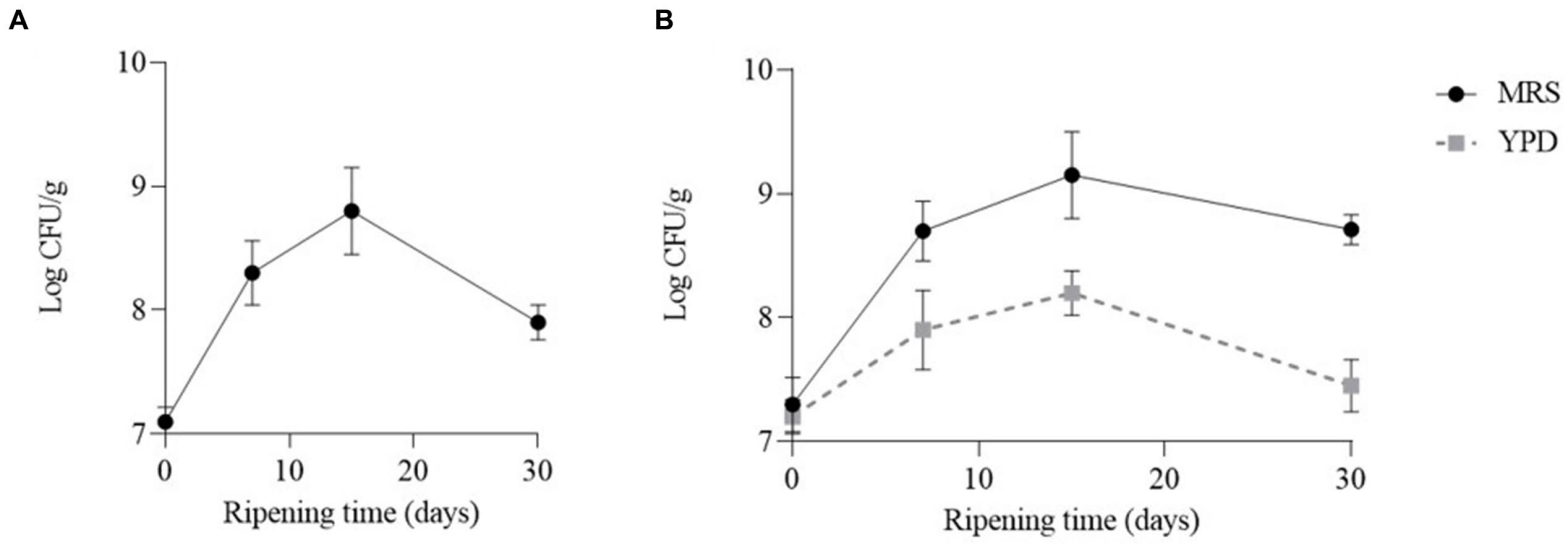
Evolution of lactic acid bacteria and yeasts in the cheeses obtained with CC **(A)** and CC + FM09 **(B)**. Data are means ± standard deviations of 3 independent experiments, each carried out in triplicate.

In both cheeses the number of LAB increased during the first 15 days and then decreased. In particular, the number of LAB reached a value of 8.8 Log CFU/g and 9.15 Log CFU/g, in cheeses obtained with CC and CC + FM09, respectively. After 30 days of ripening the number of LAB was about a Log higher in the cheeses obtained with the co-culture (8.71 Log CFU/g) than in the others (7.92 Log CFU/g) (Figure 2). The number of yeasts reached the highest concentration after 15 days (8.2 Log UFC/g), and decreased after 30 days of ripening (7.45 Log CFU/g) (Figure 2). The yeasts were absent in cheeses obtained with CC.

### 3.2 Aroma compounds

A total of 55 volatile compounds were detected in the cheeses, including 10 organic acids, 13 higher alcohols, 17 esters, 7 aldehydes, and 8 ketones (Table 2). The content of higher alcohols was higher in cheeses obtained with FM09 + CC (1,079 μg/kg) than in the others (660.19 μg/kg). The most abundant were isoamyl alcohol (fusel, alcoholic, whiskey, fruity, banana), 1-octanol (waxy, green, orange, aldehydic, rose, mushroom), 2-butanol (sweet, apricot), and 2-phenylethanol (floral, sweet, rose). Their concentrations were 58.97 ± 7.23 μg/kg and 56.45 ± 5.19 μg/kg, 99.13 ± 11.57 μg/kg and 141.22 ± 0.45 μg/kg, 315.22 ± 1.45 μg/kg and 517.33 ± 2.41 μg/kg, 78.44 ± 3.22 μg/kg and 199.44 ± 12.44 μg/kg, in cheeses obtained with CC and FM09 + CC, respectively.

**Table 2:** Main volatile compounds detected in cheeses after 30 days of ripening.

Compounds	OTS (μg/kg)	Odor description	Concentration (μg/kg)		OAV	
Organic acids			CC	FM09 + CC	CC	FM09 + CC
Acetic acid	124*	Sharp, pungent, sour, vinegar	93.32 ± 23.22^A^	167.17 ± 22.11^B^	1.08	1.35
Butanoic acid	175*	Sharp, acetic, cheesy, buttery, fruity	193.44 ± 14.33^A^	223.31 ± 15.66^A^	1.11	1.28
Hexanoic acid	3000*	Sour, fatty, sweaty, cheesy	84.22 ± 18.22^A^	95.33 ± 4.33^A^	–	–
Octanoic acid	3000*	Fatty, waxy, rancid, oily, vegetable, cheesy	35.44 ± 5.44^A^	91.33 ± 7.22^B^	–	–
Decanoic acid	10000*	Rancid, sour, fatty, citrus	93.33 ± 5.44^A^	143.44 ± 12.54^B^	–	–
Pentanoic acid	3000**	Acidic, sharp, cheesy, sour, milky, tobacco, fruity	3.42 ± 0.17^A^	23.44 ± 7.44^B^	–	–
Nonanoic acid	3000**	Waxy, dirty, cheesy, dairy	1.55 ± 0.78^A^	1.66 ± 0.44^A^	–	–
Dodecanoic acid	10000*	Fatty, coconut, bay	11.33 ± 0.67^A^	13.44 ± 0.56^A^	–	–
2-ethyl-butanoic acid	n.f.	Acidic, fruity, whiskey, dry, berry	8.44 ± 4.45^A^	9.56 ± 5.66^A^	–	–
3-methyl-butanoic acid	n.f.	Sour, sweaty, cheesy, tropical	2.11 ± 0.34^A^	3.44 ± 0.89^A^	–	–
Total			566.6	772.12		
Higher alcohols						
1-pentanol	4000*	Pungent, bready, yeasty, fusel, winey, solvent	9.31 ± 3.44^A^	14.33 ± 4.56^A^	–	–
2-butanol	500**	Sweet, apricot	315.22 ± 1.44^A^	517.33 ± 2.41^B^	–	1.03
2-nonanol	50**	Waxy, green, creamy, citrus, orange, cheesy, fruity	19.56 ± 2.19^A^	61.44 ± 1.78^B^	–	1.51
2-petanol	n.f.	Alcoholic, fermented musty, sweet, winey, banana	10.22 ± 2.78^A^	15.32 ± 2.44^A^	–	–
3-methyl-1 pentanol	n.f.	Pungent, fusel, cognac, winey, cocoa, green, fruity	n.d.	0.55 ± 0.22	–	–
2-methyl-1-hexanol	n.f.	n.f.	n.d.	0.64 ± 0.11	–	–
1-octanol	110**	Waxy, green, orange, aldehydic, rose, mushroom	99.13 ± 11.57^A^	141.22 ± 0.45^B^	–	1.28
1-butanol	500*	Fusel, oily, sweet, balsamic, whiskey	1.43 ± 0.24^A^	5.44 ± 0.15^B^	–	–
2-heptanol	3**	Fresh, lemongrass, herbal, sweet, floral, fruity, green	2.33 ± 0.34^A^	7.13 ± 0.56^B^	–	2.37
2–3 butanediol	3***	Fruity, creamy, buttery	n.d.	4.34 ± 0.77	–	1.45
1.2-propanediol	n.f.	Slight alcoholic	0.11 ± 0.04^A^	0.15 ± 0.05^A^	–	–
2-phenylethanol	140****	Floral, sweet, rose	78.44 ± 3.22^A^	199.44 ± 12.44^B^	–	1.42
Isoamyl alcohol	250**	Fusel, alcoholic, fruity, banana	58.97 ± 7.23^A^	256.45 ± 5.19^B^		1.02
1-hexanol	2500**	Pungent, fusel, oily fruity, alcoholic, sweet, green	n.d.	1.34 ± 0.54	–	–
Total			594.72	1224.97		
Esters						
Ethyl acetate	5000*	Ethereal, fruity, sweet, herbal, green	13.45 ± 1.33^A^	97.22 ± 2.45^B^	–	–
Ethyl benzoate	60**	Fruity, dry, musty, sweet, wintergreen	38.22 ± 0.55^A^	88.12 ± 0.17^B^	–	1.47
Ethyl butanoate	1*	Fruity, juicy, pineapple, cognac	88.12 ± 2.71^A^	156.55 ± 5.44^B^	88.12	156.55
Ethyl decanoate	530***	Sweet, waxy, fruity, apple, grape, oily brandy	n.d.	5.66 ± 1.33	–	–
Ethyl heptanoate	2.2**	Fruity, pineapple, cognac, rummy, winey	n.d.	2.71 ± 1.03	–	1.23
Ethyl hexanoate	1**	Sweet, fruity, pineapple, waxy, green banana	65.71 ± 1.33^A^	81.78 ± 4.55^B^	65.71	81.78
Ethyl nonanoate	50***	Fruity, rose waxy, rummy, winey, natural, tropical	41.79 ± 5.69^A^	61.22 ± 0.45^B^	–	1.22
Ethyl octanoate	194****	Fruity, winey, waxy sweet, apricot, banana, pear	105.94 ± 1.89^A^	197.44 ± 15.44^B^	–	1.01
Diethyl succinate	n.f.	Fruity, apple, cooked, apple, ylang	n.d.	8.47 ± 1.33	–	–
Ethyl lactate	14000**	Sharp, tart, fruity, buttery, butterscotch	31.56 ± 0.89^A^	72.78 ± 4.78^B^	–	–
Ethyl laurate	n.f.	Ethereal, fruity, green	n.d.	1.45 ± 0.34	–	–
Ethyl valerate	n.f.	Sweet, fruity, apple, pineapple, green, tropical	n.d.	2.33 ± 0.17	–	–
Isoamyl lactate	67****	Fruity, creamy, nutty	n.d.	4.93 ± 0.21	–	–
Isopropyl isobutanoate	n.f.	Pineapple, sweet, fruity, citrus, pear	n.d.	5.44 ± 0.43	–	–
Phenylethyl acetate	19****	Sweet, floral-rose, peach, honey, green	17.69 ± 0.12^A^	51.78 ± 0.33^B^	–	2.72
Isoamyl acetate	2**	Sweet, fruity, banana, solvent	1.97 ± 0.18^A^	91.13 ± 1.56^B^	–	45.57
3-methylbutyl butanoate	n.f.	Fruity, green, apricot, pear, banana	n.d.	0.89 ± 0.11	–	–
Total			404.45	929.9		
Aldehydes						
2-methyl butanal	n.f.	Musty, cocoa, coffee, nutty, malty	5.33 ± 0.45^A^	4.44 ± 0.34^A^	–	–
3-methyl butanal	n.f.	Ethereal, aldehydic, chocolate, peach, fatty	2.71 ± 0.14^A^	2.11 ± 0.23^A^	–	–
Benzaldehyde	3500**	Sharp, sweet, bitter, almond, cherry	1.18 ± 0.13^A^	1.98 ± 0.34^A^	–	–
Hexanal	4.5*	Fresh, green fatty, aldehydic, grassy, fruity, sweaty	5.22 ± 1.45^A^	6.45 ± 2.22^A^	1.16	1.43
Octanal	0.7**	Aldehydic, waxy, citrus, orange, green, fresh fatty	0.98 ± 0.11^A^	1.33 ± 0.78^A^	1.4	1.90
Pentanal	12*	Fermented, bready, fruity, berry	9.11 ± 0.09^A^	11.54 ± 1.45^A^	–	–
Nonanal	1*	Waxy, aldehydic, rose, fresh, orange, fatty	7.56 ± 1.33^A^	7.33 ± 1.89^A^	7.56	7.33
Total			32.09	35.18		
Ketones						
Acetoin	14***	Sweet, buttery, creamy, dairy, milky, fatty	56.34 ± 3.67^A^	77.44 ± 5.44^B^	4.02	4.82
Diacetyl	6.5**	Buttery, sweet, creamy, pungent, caramellic	19.67 ± 0.89^A^	21.77 ± 0.34^A^	3.02	3.35
2-propanone	500000**	Solvent, ethereal, apple, pear	1.88 ± 1.78^A^	2.43 ± 1.22^A^	–	–
2-butanone	500000*	Ethereal, fruity, camphoreous	11.14 ± 1.44^A^	19.11 ± 0.55^B^	–	–
2-pentanone	70000**	Sweet, fruity, ethereal, winey, banana, woody	2.36 ± 1.12^A^	4.24 ± 0.18^B^		–
2-heptanone	140*	Fruity, spicy, sweet, herbal, coconut, woody	16.22 ± 1.78^A^	15.33 ± 2.56^A^	–	–
2-nonanone	5*	Fresh, sweet, green, earthy, herbal	6.35 ± 0.76^A^	14.44 ± 1.91^B^	1.24	2.89
2-undecanone	10***	Waxy, fruity, creamy, fatty, floral	10.72 ± 1.87^A^	18.33 ± 2.78^B^	1.07	1.83
Total			124.68	173.09		

Different letters in the same line indicate significant differences (*p* < 0.05, FDR corrected). Data are means ± standard deviations of 3 independent experiments, each carried out in triplicate. *Sacchi et al. (2020), **http://www.leffingwell.com/odorthre.htm, ***Yang et al. (2024), ****Tsouli Sarhir et al. (2022).

The inoculation of *K. marxianus* induced an increase of esters’ content. In both cheeses ethyl esters were more abundant than acetate ones. These compounds were mainly present in cheeses obtained with CC + FM09. The main esters detected were ethyl butanoate (88.12 ± 2.71 μg/kg and 156.55 ± 5.44 μg/kg in cheeses obtained with CC and FM09 + CC, respectively), ethyl hexanoate (65.71 ± 1.33 μg/kg and 81.78 ± 4.55 μg/kg in cheeses obtained with CC and FM09 + CC, respectively), ethyl octanoate (105.94 ± 1.89 μg/kg and 197.44 ± 15.44 μg/kg in cheeses obtained with CC and FM09 + CC, respectively).

Acetic (93.32 ± 23.22 μg/kg and167.17 ± 22.11 μg/kg in cheeses obtained with CC and FM09 + CC, respectively) and butanoic acids (193.44 ± 14.33 μg/kg and 223.31 ± 15.66 μg/kg in cheeses obtained with CC and FM09 + CC, respectively) were the main acids detected, followed by hexanoic (84.22 ± 18.22 μg/kg and 95.33 ± 4.33 μg/kg in cheeses obtained with CC and FM09 + CC, respectively), and decanoic (93.33 ± 5.44 μg/kg and 143.44 ± 12.54 μg/kg in cheeses obtained with CC and FM09 + CC, respectively) acids.

Aldehydes were present in similar concentration in both cheeses (32.09 μg/kg in CC cheeses and 35.18 μg/kg in FM09 + CC cheeses). The main aldehydes detected were 2-methylbutanal (5.33 ± 0.45 μg/kg and 4.44 ± 0.34 μg/kg in cheeses obtained with CC and FM09 + CC, respectively), hexanal (5.22 ± 1.45 μg/kg and 6.45 ± 2.22 μg/kg in cheeses obtained with CC and FM09 + CC, respectively), and nonanal (7.56 ± 1.33 μg/kg and 7.33 ± 1.89Aμg/kg in cheeses obtained with CC and FM09 + CC, respectively).

The inoculation of *K. marxianus* FM09 influenced the content of ketones, in fact the cheeses made only with CC had a ketone content approximately 50 μg/kg lower than the others. The main ketones detected were acetoin (56.34 ± 3.67 μg/kg and 77.44 ± 5.44 μg/kg in cheeses obtained with CC and FM09 + CC, respectively) and diacetyl (19.67 ± 0.89 μg/kg and 21.77 ± 0.34 μg/kg in cheeses obtained with CC and FM09 + CC, respectively).

**Figure 3:**
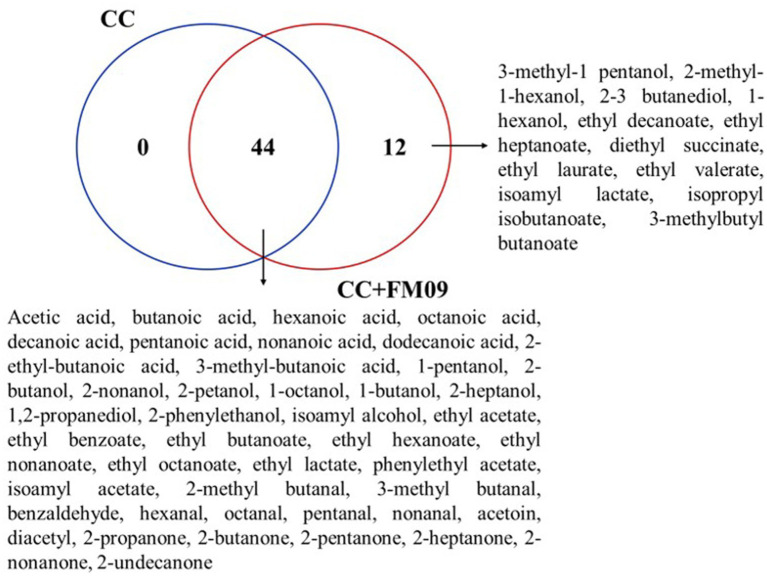
Venn diagram showing the similarities and differences of volatile compounds between cheese samples The Venn diagram was generated with the web tool provided by the Bioinformatics and Systems Biology of Gent.

The Venn diagram combined with the calculation of OAV method can explore the unique volatile compounds of cheeses obtained with CC and CC + FM09. The Venn diagram showed that the cheeses shared 44 compounds, while 12 were found only in cheeses obtained with CC + FM09 (Figure 3). This data suggested a specific effect of the inoculated yeast in the definition of cheese aroma; and this effect was particularly evident for esters. In fact, 8 esters out of 17 were specifically associated to the presence of *K. marxianus* FM09.

The contribution of aroma is not only determined by the concentration but also by the odor threshold values. Therefore, the OAV were calculated to further analyze the contributions of aroma-active compounds (Table 2). Cheeses obtained with CC + FM09 showed a richer flavor than the control. In fact, only 11 compounds showed OAV > 1 in cheeses obtained with CC, and 24 in those obtained with CC + FM09. Moreover, OAV were higher in these last cheeses. This result suggested once again the effect of *K. marxianus* on cheese aroma profile. The main differences were detected for higher alcohols, and esters. In particular, 2-butanol, 2-nonanol, 1-octanol, 2-heptanol, 2,3-butanediol, 2-phenylethylalcohol and isoamyl alcohol showed OAV >1 only in cheeses produced with FM09 + CC. Two (ethyl butanoate and ethyl hexanoate) and 8 (ethyl benzoate, ethyl butanoate, ethyl heptanoate, ethyl hexanoate, ethyl nonanoate, ethyl octanoate, phenylethyl acetate, and isoamyl acetate) esters showed OAV > 1 in cheeses produced with CC and FM09 + CC, respectively. The majority of compounds with OAV > 1 detected in cheeses obtained with CC + FM09 showed fruity notes, and the main contributors were ethyl butanoate, ethyl hexanoate, and isoamyl acetate. The evaluation of aroma characteristics other than fruity, showed that cheeses produced with FM09 + CC showed higher buttery notes than the others, due to the contribution of acetoin, diacetyl and 2,3-butanediol. Moreover, these cheeses were also characterized by green and fresh aroma thanks to the OAV of 2-heptanol, hexanal, octanal, 1-octanol, 2-nonanol.

### 3.3 qPCR analysis

**Figure 4:**
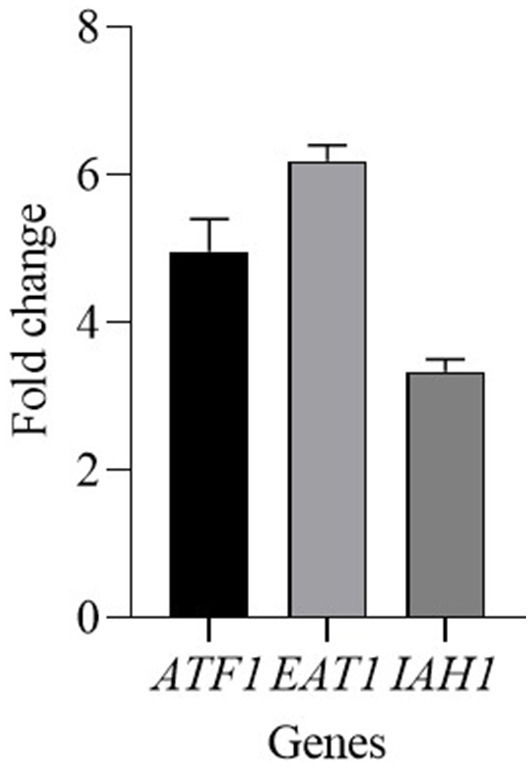
Relative transcript levels of genes involved in ester content regulation in *K. marxianus* after 30 days of ripening of cheeses made with CC + FM09. Transcript levels are expressed as x-fold increase in comparison with the expression at T0. Three biological replicates were performed, with 3 technical repeats.

*Kluyveromyces marxianus* mainly influenced the content of esters, therefore the expression of some genes involved in their production was analyzed. Since the activity of ester-synthesizing enzymes and ester-hydrolyzing enzymes are related to the ester accumulation the following genes were tested: *ATF*1 and *EAT*1 genes encode for alcohol acetyl-transferases (AATase), and *IAH*1 gene for an esterase. *ATF*1 and *EAT*1 genes were upregulated and showed a fold change of 4.96 and 6.19, respectively (Figure 4). The gene *IAH*1 was upregulated, with a fold change of 3.34.

## 4 Discussion

This study investigated the effect of *K. marxianus* FM09 on cow cheese characteristics, and the metabolic potential of this strain to impact cheese flavor. The inoculation of FM09 induced an increase of pH, probably because of the lactic acid utilization by this yeast, and favored the proteolysis. A similar result concerning pH was obtained by Pisano et al. (2020) when *K. lactis* was used as adjunct culture for Caciotta cheese. *K. marxianus* participates in deacidification of the curd through lactate/lactose consumption with a notable increase in pH that enables the acid-sensitive bacteria to develop at the cheese surface (Zheng et al., 2021). The higher proteolysis detected in cheeses made with FM09 + CC is in agreement with Xiao et al. (2020) who used *K. marxianus* A2 as adjunct culture for the production of Kazak cheeses. The authors highlighted that this yeast contributed to the formation of FAAs, in fact the cheeses showed significant higher amounts of amino acids, except for valine and leucine, than the other cheeses obtained with *Pichia fermentans* A19 and *P. kudriavzevii* A11. The proteolytic activity of *K. marxianus*, due to peptidases like aminopeptidases and carboxypeptidases, is well known and could result in great amounts of volatile compounds, contributing, thus, to the development of aroma and flavor of cheese (Tofalo et al., 2014; Xiao et al., 2020). Moreover, it should be postulated a synergistic effect between *K. marxianus* and LAB resulting in stronger proteolysis. A similar effect has been observed between *D. hansenii* and LAB (Ferreira and Viljoen, 2003). The highest proteolysis detected in these cheeses can also explain the increase of pH. In fact, the acids can be neutralized by some alkaline substances from protein decomposition, which will cause the increase of pH (Gori et al., 2007). The other parameters were similar to those obtained for mountain Caciotta cheese (Cardin et al., 2024).

The microbiological analyses revealed that *L. casei* reached a higher cell concentration in presence of *K. marxianus* FM09. Similar results were obtained by Huang et al. (2020) who noticed that the population of total LAB was significantly higher in multi-starters fermentation system (*Streptococcus thermophilus* + *K. marxianus* and *Lactobacillus delbrueckii* spp. *bulgaricus* + *K. marxianus*) (log 9.65 CFU/mL) than single culture (log 9.45 CFU/mL). Probably the metabolites produced by yeast decomposition of the substrate such as amino acids and vitamins are used by LAB to further promote growth (Zhang et al., 2017). Meanwhile, some LAB metabolites also provide an energy source for yeast growth (Zhang et al., 2017). In this study, the combination of this species/strains was useful for the production cow cheeses since non-competitive or inhibitory effects between these 2 strains were detected.

The inoculation of *K. marxianus* influenced the volatile profile of cheeses. In fact, the cheeses obtained with FM09 + CC showed qualitative and quantitative differences if compared to cheeses obtained only with CC. The content of organic acids was increased of 37%, and the main acids interested were acetic, butanoic, octanoic, decanoic and pentanoic ones (Table 2). Carboxylic acids can be originated from three main biochemical pathways: (1) lipolysis (hydrolysis of triglycerides into free fatty acids, such as butanoic, pentanoic, hexanoic, heptanoic, octanoic, nonanoic, decanoic and undecanoic acids); (2) proteolysis (breakage of caseins into peptides and amino acids, such as 2-methylpropanoic and 3-methylbutanoic acids); and (3) lactate metabolism (acetic and propanoic acids) (Delgado et al., 2011).

*Kluyveromyces marxianus* is known to produce a variety of organic acids. These include acetic acid, propionic acid, butanoic acid, octanoic acid, among others (Pentjuss et al., 2017; Fasoli et al., 2015; Cheon et al., 2014). The highest increase was recorded for octanoic acid (160%) and acetic acid (79%). Acetic and butanoic acids contributes to the typical flavor of most types of cheese, giving rise to vinegar-like, fruity, and sharp odors (Zheng et al., 2017). In particular, butanoic acid has a rancid cheese-like odor and plays an important role in the flavor of many cheese types such as Camembert, Cheddar (aged, regular and low fat), Grana Padano, Gruyere, Pecorino, Ragusano and Roncal cheese (Curioni and Bosset, 2002). Hexanoic and decanoic acids have been detected in Grana Padano and Roncal cheeses (Curioni and Bosset, 2002). The increase detected in this study is in agreement with previous observations. Acetic acid is the main organic acid produced by *K. marxianus*, in fact its accumulation during exponential growth phase is typical for *K. marxianus* fermentations (Pentjuss et al., 2017; Löser et al., 2013; Signori et al., 2014). The production of octanoic acid has been already reported by other authors (Zheng et al., 2017; Fasoli et al., 2015). In particular, Fasoli et al. (2015) detected an increase of octanoic acid concentration when *K. marxianus* was grown in raw cheese whey (the aqueous part of milk that remains after the separation of the curd during cheese making) containing 4.8% lactose, 1.2% proteins and 0.75% fats.

The presence of *K. marxianus* induced an increase of alcohol content of 63%. All detected alcohols were more present in cheeses obtained with CC + FM09; and this increase was particularly evident for 2-phenylethanol (109%). *Kluyveromyces marxianus* is able to produce various alcohols. For instance, it has been utilized for ethanol production from crude whey, or furfuryl alcohol from furfural (Gethins et al., 2015). Moreover, alcohol dehydrogenases from *K. marxianus* have been investigated, showing the yeast’s capability to produce different alcohols with various chain lengths (Liang et al., 2014). The production of 2-phenylethanol has been largely demonstrated in *K. marxianus* (Fasoli et al., 2015; Perpetuini et al., 2022; Gethins et al., 2015; Alonso-Vargas et al., 2022). This compound is produced from the catabolism of L-phenylalanine via the Ehrlich pathway or by *de novo* synthesis from sugars, through the Shikimate pathway (Wittmann et al., 2002). Some studies demonstrated that cheese whey represented a good carbon source for 2-phenylethanol production by *K. marxianus* (Alonso-Vargas et al., 2022; Fasoli et al., 2015; Han et al., 2024). In particular, Han et al. (2024) demonstrated that co-culture of *Pediococcus lactis* and *K. marxianus* improved the metabolic capacity for producing 2-PE. This may be because *P. lactis* effectively activated the Ehrlich pathway of *K. marxianus* and enhanced the robustness of *K. marxianus* through improving the fluidity and stability of cell membranes.

The ethyl esters were the main esters detected in this study in both cheeses. They are considered to be major contributors to overall flavor and were the most abundant esters in Grana Padano cheese (Moio and Addeo, 1998), in Brie cheese (Karahadian et al.,1985), in bovine Mozzarella cheese (Moio et al., 1993), and in Parmesan cheese (Nursten, 1997), and could contribute to the aroma of cheese by minimizing the sharpness and bitterness derived from carboxylic acids (Curioni and Bosset, 2002). The use of *K. marxianus* induced an increase of esters content of 174%. All esters showed a higher concentration in cheeses obtained with CC + FM09 than in the others. This result is in agreement with the recognized ability of *K. marxianus* to produce esters (Morrissey et al., 2015; Reyes-Sánchez et al., 2019). The most common esters produced by *K. marxianus* are 2-phenylethyl acetate, isoamyl acetate, and ethyl acetate. These esters are synthesized from 2-phenylethanol, isoamyl alcohol, and ethanol, respectively (Morrissey et al., 2015). Their content showed the highest percentage increase in cheeses obtained with *K. marxianus*. This data is in agreement with the content of higher alcohols detected. In fact, according to Morrissey et al. (2015) the conditions that give rise to elevated levels of higher alcohols thus also provide the circumstances in which elevated levels of acetate esters are possible. The qPCR analysis was applied to better demonstrate the influence of *K. marxianus* FM09 on esters content. The increased fold change detected for *ATF*1 and *EAT*1 genes is in agreement with previous observations which showed that their deletion induced a reduction of ethyl acetate, isoamyl acetate, and 2-phenylethyl acetate (Löbs et al., 2018). The highest expression of *EAT*1 gene is in agreement with Löbs et al. (2018) who revealed the key role played by this gene in *K. marxianus* esters production. In fact, its deletion caused a reduction of esters content of more than 80%. Concerning the over expression of *IAH*1 gene, it should be noted that the role of *IAH*1 gene in ester metabolisms is not clear. In fact, some authors supposed that *IAH*1 could have an ester synthase activity (Vidal et al., 2013; Löser et al., 2014; Löbs et al., 2017; Muñoz-Miranda et al., 2024; Dank et al., 2018). In particular, Dank et al. (2018) hypothesize that *IAH*1 gene may have promiscuous activity on ethyl esters, resulting in a redirection of intracellular fluxes of amino acid catabolism intermediates to ethyl esters. Moreover, it should be noted that the accumulation of esters relies on the balance between esters synthesis and degradation (van Rijswijck et al., 2019). Dank et al. (2018) showed that the deletion of the *IAH*1 and *TIP*1 genes in *S. cerevisiae* results in an intracellular metabolite imbalance, triggering a decrease in ester substrates, a reduction in ester synthase activity to compensate for the decreased hydrolyzing activity, or an upregulation of promiscuous esterases.

The inoculation of *K. marxianus* favored the accumulation of ketones in the cheese. Ketones are considered key component of the flavor of several cheeses (Nogueira et al., 2005) and are generally produced by the enzymatic oxidative decarboxylation of fatty acids by LAB. The main ketones detected were acetoin and diacetyl. Acetoin can be derived from diacetyl metabolism (Rehman et al., 2000) or from pyruvate metabolism during the conversion of lactose to lactic acid (Nogueira et al., 2005). It is characterized by buttery notes, and has been found in several cheeses above the aroma threshold (Sablé and Cottenceau, 1999). Diacetyl is obtained from pyruvate stemming from lactose and citrate metabolism. It is appreciated for its buttery and nut-like notes and was identified as a key aroma component of Camembert, Cheddar and Emmental (Curioni and Bosset et al., 2002). *K. marxianus* ability to produce ketones has been shown also by Risner et al. (2019). These authors found differences in ketones production by *K. marxianus* between acid and sweet whey distillates. Sweet whey distillate contained significantly greater concentration of ketones than the others, and 2-Heptanone, 2-pentadecanone, 2-tridecanone, and 2-undecanone were identified exclusively in these distillates. Perpetuini et al. (2022) reported that the production of ketones was influenced by K*. marxianus* aggregation state, and they were mainly produced by sessile cells and the main ketones detected were 2-nonanone, and 8-hydroxyoctan-2-one.

Aldehydes are transitory compounds in cheese because they are rapidly reduced to primary alcohols or even oxidized to the corresponding acids (Curioni and Bosset, 2002). However, they can have an important impact on cheese aroma since they have low threshold values. It should be noted that their contribution to cheese aroma depends not only on the concentration of single compound, since a synergistic effect can be also present. For example, 2-methylbutanal and 3-methylbutanal, both found in this study, can have a synergistic effect. 2-methylbutanal produced malty, cacao, and apple-like aromas, while 3-methylbutanal produced malty, coffee, and cacao aromas. Therefore, the mixture of these compounds emitted a pleasant nutty and malty odor with a threshold of 29.29% of the single compound thresholds (Chen et al., 2020). Both cheeses were characterized by the presence of pentanal, hexanal, and nonanal. These aldehydes are commonly found in cheeses (Grana Padano, Mozzarella, among others) and confer green grass and herbaceous aromas (Curioni and Bosset, 2002). Such compounds may also result from *β*-oxidation of unsaturated fatty acids or even light-induced reactions, although such reactions are not likely to occur in cheese (Borle et al., 2002). *K. marxianus* can produce these compounds as part of its metabolic processes for xylitol, ethanol, lactate, and aroma compounds production (Baptista and Domingues, 2022). Additionally, the *YGL157*w gene product of *Kluyveromyces marxianus* DMB1 strain has been characterized as a broad specificity NADPH-dependent aldehyde reductase, indicating its involvement in aldehyde metabolism (Akita et al., 2015). The obtained results suggested a synergistic effect between *K. marxianus* and *L. casei* in terms of aroma compounds production. A synergistic effect between *K. marxianus* and LAB (*L. helveticus* and *L. delbrueckii* subsp. *bulgaricus*) concerning the increase of lactic acid production has been already reported especially (Plessas et al., 2008; Lee, 2005). This positive effect between yeast and LAB could be due to the fact that yeasts provide LAB with growth factors such as vitamins that promote growth and consequently leading to increased lactic acid production, while the yeasts uses the bacterial end-products of LAB as energy sources (Plessas et al., 2008).

This study established the effect of *K. marxianus* FM09 in flavor development and gross composition of short-aged cow cheese. The inoculation of this yeasts induced an increase of esters, higher alcohols, organic acids, and ketones, as well as the production of key volatile compounds was observed. These results demonstrated the potential of *K. marxianus* FM09 as co-adjunct culture even if further studies are necessary to better investigate the interactions of the strains used and the cheese microbiota. The metabolic pathways of *K. marxianus* involved in the definition of cheese characteristics should be highlighted using multi -omics approaches.

## Data Availability

The original contributions presented in the study are included in the article/supplementary material, further inquiries can be directed to the corresponding author.
